# Is there an association between 30-day mortality and adrenaline infusion rates in post-ROSC patients? A retrospective observational analysis

**DOI:** 10.29045/14784726.2022.12.7.3.1

**Published:** 2022-12-01

**Authors:** Peter Owen, Martyn Sherriff

**Affiliations:** South East Coast Ambulance Service NHS Foundation Trust; University of Bristol

**Keywords:** adrenaline, infusion, out-of-hospital cardiac arrest, post-cardiac arrest syndrome, ROSC, vasopressors

## Abstract

**Introduction:**

Revised guidelines for the management of cardiac arrest have placed greater emphasis on early defibrillation and closed chest compressions; subsequently there has been a significant rise in the number of patients gaining a return of spontaneous circulation (ROSC). As a consequence, emergency medical services have realised the importance of therapies delivered during this phase of care. In some Trusts this includes the use of inotropic agents to augment the cardiovascular system and maintain adequate cerebral and coronary perfusion pressures to mitigate the effects of post-cardiac arrest syndrome. Currently, limited evidence exists with regards to the efficacy of such treatments in the pre-hospital phase.

**Methods:**

Retrospective observational analysis of out-of-hospital cardiac arrest patients who received an adrenaline infusion by critical care paramedics. Infusion rates, time of call (ToC) to ROSC and 30-day mortality were compared.

**Results:**

Over a 2-year period, 202 patients were recorded as having an adrenaline infusion commenced. Of these, 25 were excluded as they did not meet criteria or had incomplete data and 22 were excluded as the infusion was stopped at scene; 155 patients were admitted to hospital. There were no survivors in the non-shockable group and three survivors in the shockable group at 30 days. A rare events analysis found no relationship between infusion rate, ToC to ROSC and 30-day mortality (Wald chi2, 1.37).

**Conclusion:**

Commencement of adrenaline infusions in post-ROSC was associated with significant 30-day mortality, especially in non-shockable rhythms. Further research is needed to elucidate whether this intervention has any benefit in the post-ROSC patient.

## Introduction

**Figure 1:**
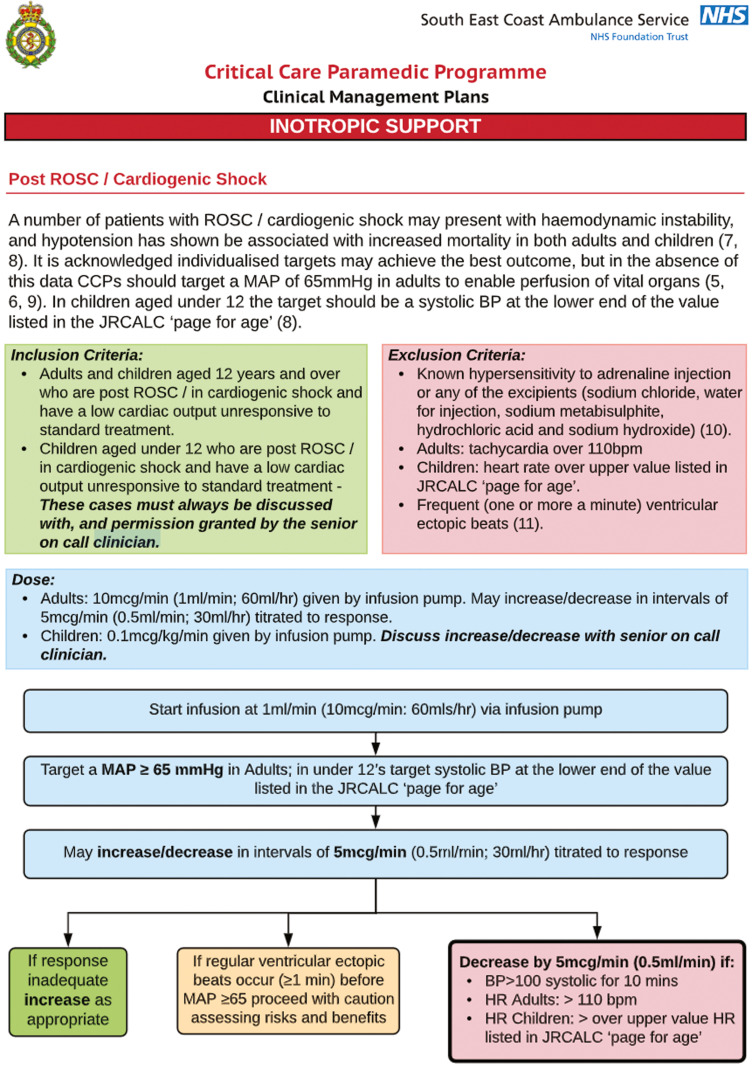
Figure 1. Clinical management plan for inotropic support.

Source: South East Coast Ambulance Service NHS Foundation Trust (2018).

Changes to resuscitation guidance provided by the European Resuscitation Council have underlined the importance of external chest compressions and defibrillation during cardiac arrest (Soar et al., 2015). This has resulted in greater numbers of patients who gain a return of spontaneous circulation (ROSC) (Larribau et al., 2018). As a consequence, emergency medical services are placing a greater emphasis on care provided during post-ROSC care, and it is now included in the chain of survival (Handley et al., 2005). Critical care paramedics (CCPs) from the South East Coast Ambulance Service (SECAmb) utilise an infusion pump that delivers a continuous infusion of adrenaline to provide inotropic support in post-ROSC patients (South East Coast Ambulance Service NHS Foundation Trust, 2018). The clinical management plan SECAmb CCPs use for inotropic adrenaline is shown in Figure 1. The benefits of infusion include the ability for the inotrope to remain in therapeutic range and avoid the peaks and troughs normally associated with bolus dosing (Chambers, 2019), as well as avoiding any errors associated with the preparation and administration of push-dose pressors (Cole, 2018).

Despite this routine use of adrenaline during the post-ROSC period, there is a paucity of information regarding the use of adrenaline in this phase, especially in the pre-hospital environment. The recent publication of the PARAMEDIC2 (Perkins et al., 2018) trial has demonstrated improved ROSC rates (unadjusted odds ratio (UOR) 1.39, 95% confidence interval (CI) 1.06–1.82) but no improvement in neurological outcomes (UOR 1.18, 95% CI 0.86–1.61) in patients randomised to receive adrenaline intra-arrest. The beneficial alpha-1 effects of adrenaline have been demonstrated to improve cardiac perfusion pressures, but are known to conversely reduce cerebral blood flow and thus increase areas of ischaemia in the brain parenchyma (Mentzelopoulos & Zakynthinos, 2017). This is postulated as one of the primary mechanisms for the lack of improvement in neurological outcomes seen during the PARAMEDIC2 trial.

The management of the post-ROSC patient is complicated due to the number of poorly understood underlying pathological processes and is defined in the literature as post-cardiac arrest syndrome (PCAS) (Neumar et al., 2008). PCAS is described as comprising four main components: anoxic brain injury, arrest related myocardial dysfunction, systemic ischemic/reperfusion response and persistent precipitating pathology. How we mitigate PCAS appears to be of great importance, as adrenaline has been found to impair cerebral microcirculatory flow and potentially worsen long-term outcomes (Gough & Nolan, 2018). There is some evidence to support other pharmacotherapeutics such as norepinephrine that prevent the microcirculatory shutdown due to reduced alpha-1 effects (Levy et al., 2018; Myburgh et al., 2008).

It is the goal of this study to examine whether there is a mortality benefit associated with increased adrenaline infusion rates in the post-ROSC patient group. The null hypothesis is that increased adrenaline infusion rates will have little to no benefit in the post-ROSC patient.

## Methods

The primary end point of the study was the comparison of adrenaline infusion rates with 30-day mortality and this took the form of a retrospective chart review. Physiological data and adrenaline infusion rates from CCPBase (Medic One Systems Ltd, UK) were matched with 30-day survival data from the Trust’s cardiac arrest database. The secondary endpoint was time of call (ToC) to ROSC versus maximum infusion rate. ToC to ROSC was used as a surrogate marker for the duration of the arrest and would give an indication of exposure to lack of cardiac output. Maximum infusion rate was used, as this was an indicator of maximal sympathomimetic exposure during the patient encounter. Data were collected in Microsoft Excel (2011) and statistical analysis was performed using the statistical software Stata16^®^. Descriptive statistics were used to describe the demographic data and rare events analysis was used to ascertain if there was a correlation between ToC to ROSC time, infusion rates and 30-day mortality. Anonymisation was achieved by Trust staff removing all identifiable data which included date of birth, location, address and name of GP. Matching was achieved using CAD number and ToC. Due to the lack of survivors, it was decided that a rare events analysis utilising the Firth method of penalised maximum likelihood estimation was to be used.

### Study setting

SECAmb is a mixed urban, suburban and rural ambulance service that provides emergency ambulances to the populace of Kent, Surrey, Sussex and North East Hampshire. It receives roughly 862,000 calls a year (South East Coast Ambulance Service NHS Foundation Trust, n.d.). Of the 2700 clinical staff it employs, roughly 60 (2.2%) were operating as CCPs as of 30 April 2017.

### Participant selection

The data analysed were collected from a two-year period, between 29 March 2017 and 4 April 2019, from a customised database used by the CCPs (CCPBase, Medic One Systems Ltd, UK). Participants were patients who suffered medical out-of-hospital cardiac arrest (OOHCA) and who were in cardiac arrest on arrival of crew, who achieved ROSC and had an adrenaline infusion started by a CCP. Participants were excluded if the infusion failed to maintain ROSC to hospital or resuscitation was terminated at scene. In addition, patients < 18 years old, who were pregnant, who had suffered an arrest as a result of trauma and who were prisoners were also excluded.

## Results

During the two-year time period, 3497 OOHCA were attended by CCPs, of which a total of 202 patients received an adrenaline infusion as recorded in CCPBase data. Of these, 15 were unable to be matched with the Trust database as no 30-day mortality data existed. One was handed over intra-arrest and a further one patient was not an OOHCA. Two were excluded because trauma was the aetiological cause. Three infusions were entered incorrectly, and were in fact bolus intravenous doses. Two children (< 18 years) were excluded, and one prisoner was also excluded.

**Figure 2:**
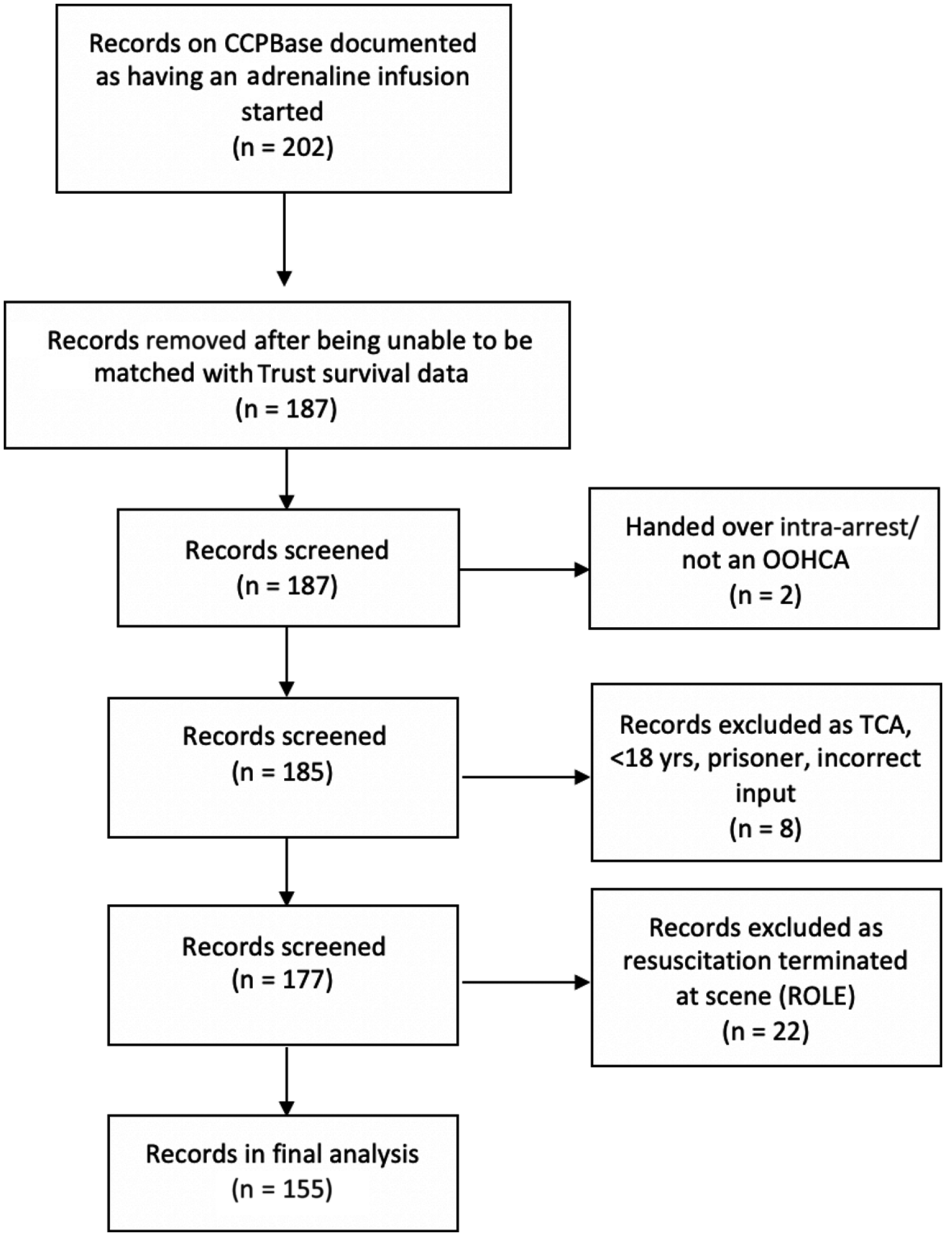
Figure 2. Participant selection.

OOHCA: out-of-hospital cardiac arrest; ROLE: recognition of life extinct; TCA: traumatic cardiac arrest.

Of the remaining 177 patients, 22 had an infusion started, but had the resuscitation attempt subsequently terminated at scene; 155 ROSC patients who had an adrenaline infusion started survived to hospital, were able to be matched to mortality data and were included in the analysis (Figure 2).

Of the 155 patients, 65 were female and 90 were male, with a mean age of 67 years (standard deviation = 15.1) (Table 1). Presenting rhythm for patients was N = 30 in a shockable rhythm (VF N = 29, VT N = 1), and N = 123 in non-shockable rhythm (asystole N = 82, PEA N = 41). There were two patients with no initial rhythm data recorded. Only three patients of the 155 survived to discharge and these all presented with VF as their initial rhythm; this is an all-cause survival rate of 1.9% in this cohort. No patients from the non-shockable group who received an adrenaline infusion survived longer than 30 days. The mean ToC to ROSC was 42.7 minutes, with a range of 12 to 115 minutes (Table 2).

**Table 1.** Demographics.

Sex	Number	Mean age (years)	Min. age (years)	Max. age (years)
Female	65	67.8	31	94
Male	90	66.8	23	89

**Table 2.** Maximum infusion rate and time of call summary statistics.

Variable	N	Missing data	Mean	SD	Min.	Max.
Max infusion rate (micrograms h-1)	154	1	714.94	312.60	100	2000
ToC to ROSC (min)	152	3	42.74	17.57	12	115

ToC: time of call; ROSC: return of spontaneous circulation; SD: standard deviation.

### Relationship between infusion rates, 30-day mortality and time of call to return of spontaneous circulation

**Figure 3:**
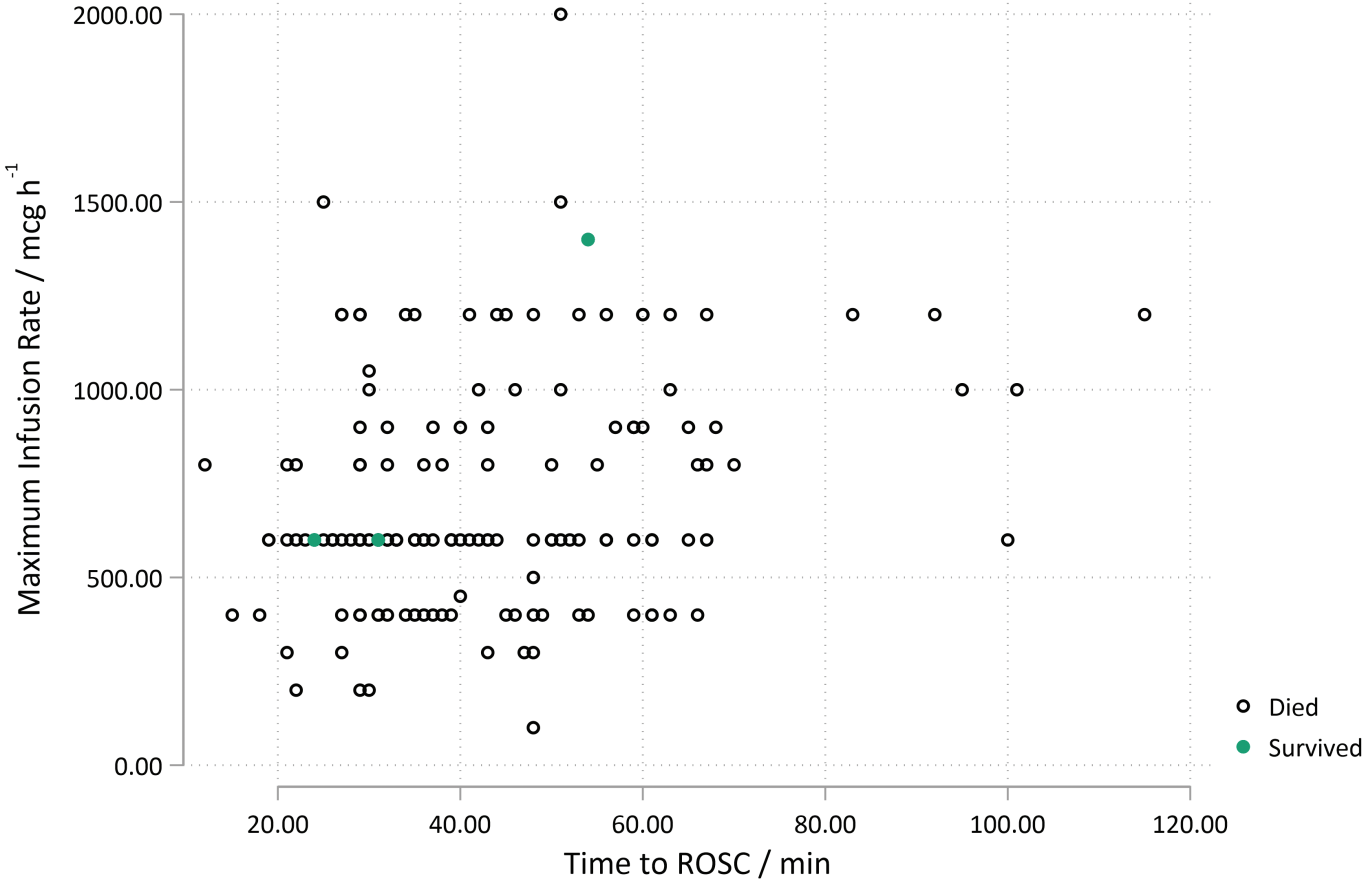
Figure 3. Relationship between maximum infusion rates, 30-day mortality and time of call to return of spontaneous circulation.

A Wald chi2 of 1.37 was found, and based on this value the data suggest there is no statistically significant effect of adrenaline infusion on survival. This is confirmed by an odds ratio of 0.95 (95% CI 0.88–1.03). Thus, the null hypothesis cannot be rejected. Figure 3 presents the three survivors as green dots. These appear in the lower values of ToC to ROSC, but this is merely an observed finding with a Pearson correlation coefficient r = 0.24.

ROSC: return of spontaneous circulation.

### Relationship between time of call to return of spontaneous circulation and maximum infusion rate

**Figure 4:**
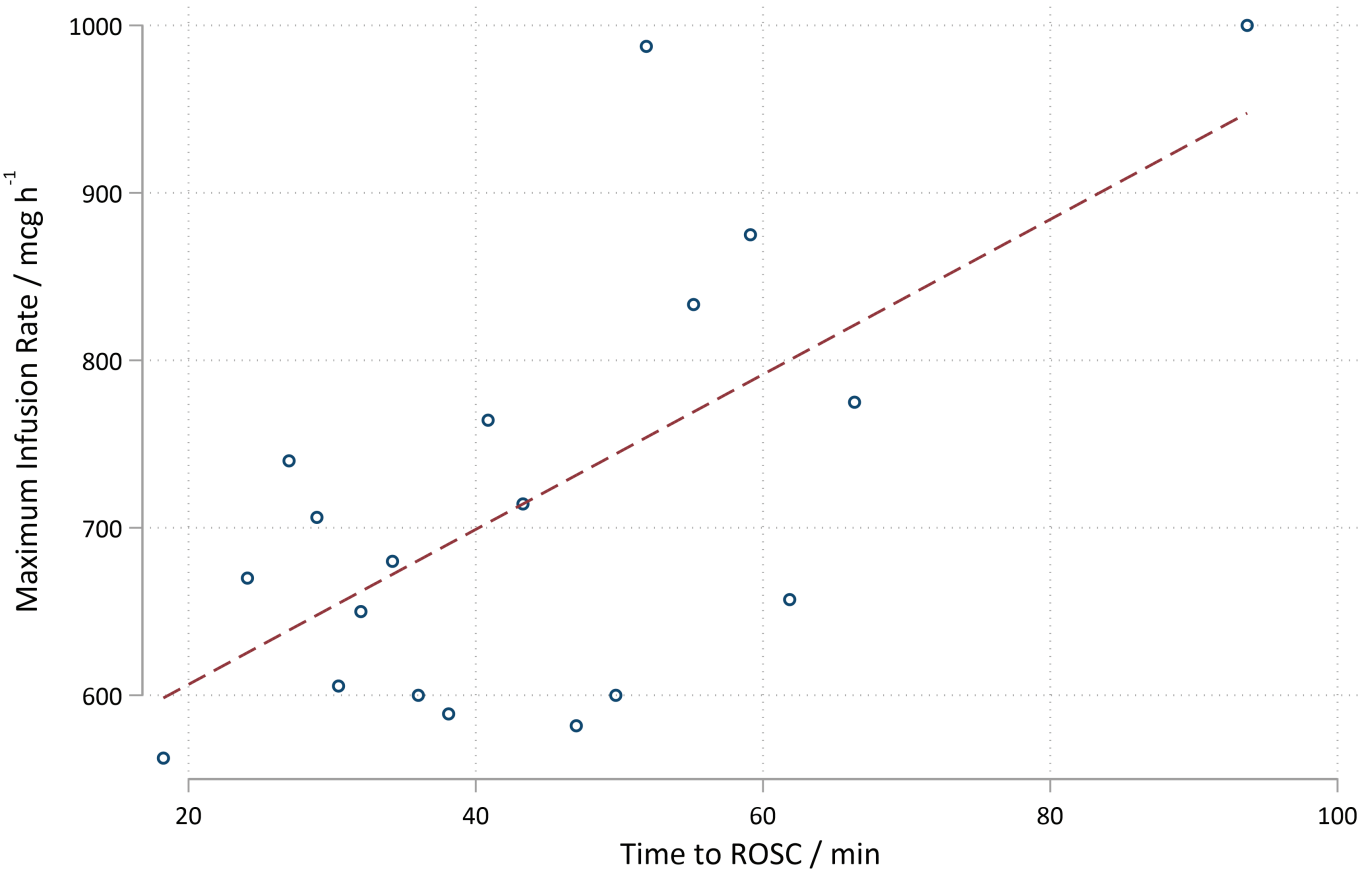
Figure 4. Graph to show relationship between maximum infusion rate and time of call to return of spontaneous circulation.

A secondary analysis on the ToC to ROSC time and the maximum infusion rate was performed using a linear regression model. A coefficient of 4.63 was found with a 95% CI of 1.85–7.405. Thus, an association between increased ToC and increased adrenaline dose is shown. The R^2^ = 0.07, which is low and reflects the large data scatter and the fact that infusion rate only takes a limited number of values (Figure 4). Although there were some deviations from normality, there is nothing to invalidate the regression.

ROSC: return of spontaneous circulation.

## Discussion

The observed effect of adrenaline infusion on 30-day mortality of post-ROSC patients appears to be of limited benefit. Due to the small size of the study group, it may have been difficult to capture any potential survivors in this cohort. This may be because non-shockable rhythms have always had a poor prognosis, with a survival to discharge rate in one study as low as 1.3% (Herlitz et al., 2008). Even in those presenting with a shockable rhythm, only 10% survived to 30 days, compared with a national picture of 27% for the same time period (Out-of-Hospital Cardiac Arrest Outcomes Project Team, 2017). This may represent a sicker patient cohort and the reason for the high 30-day mortality seen in this cohort. Studies have shown poor prognosis in patients with extended resuscitation times (> 40 mins), especially if the presenting rhythm is non-shockable (Adnet et al., 2017; Reynolds et al., 2016). In this study, the mean ToC to ROSC time was 42 minutes (42 mins = asystole; 41 mins = PEA), so it is possible that a number of these patients were likely to do poorly from the outset due to prolonged resuscitation attempts.

The linear regression shows a trend towards increasing dosages of adrenaline with increased ToC to ROSC time. It is probable that longer down-times may lead to greater tissue hypoxia and worse PCAS, which is consistent with the pathophysiological principles defined in the literature (Neumar et al., 2008). The PCAS may be so pronounced in these patients that the higher adrenaline infusion rates are required and thus are indicative of a poor prognosis from the outset of ROSC.

A greater understanding of the microcirculatory status of post-ROSC patients could avoid the negative effects associated with a broad-acting vasopressor being administered, avoiding the microcirculatory shutdown that has been associated with increased mortality (Spronk et al., 2002). Alternative pharmacotherapeutics such as noradrenaline or dobutamine may be of use, optimised using other physiological parameters such as cardiac index or systemic vascular resistance as an end point (De Backer et al., 2003). Novel diagnostic modalities like cerebral oximetry may be a better indicator of end organ perfusion when providing inotropic and vasopressor therapy (Sandroni et al., 2019). How these are delivered in the pre-hospital environment is debatable.

## Limitations

This was a retrospective review of adrenaline infusions within a small population and thus the interpretation of the results should be treated with caution. There is likely to be some selection bias as these patients do not represent the entire cohort of OOHCA attended by paramedics in this Trust, only those attended by CCPs and that received adrenaline infusions. In addition, it was assumed that the patient was in cardiac arrest at the ToC – this may or may not have been the case, which may reduce the reliability of the observed findings. Also, the 22 patients excluded due to the resuscitation attempt being stopped at scene would have likely negatively impacted the statistical results. Given that the final statistical outcome was resoundingly negative, it is likely to have had little effect on the interpretation of the results.

## Conclusion

The analysis of data from this study demonstrates there is no association between increased adrenaline infusion rates and 30-day survival. There was an association between increased ToC to ROSC and increased adrenaline infusion rates. It is likely the poor outcomes are a result of confounding by indication, and it is possible, despite maximal efforts, that these patients were always unlikely to do well. There may be inherent difficulties with using one agent to support and maintain adequate cerebral and coronary perfusion pressures. Paradoxically, the macrovascular targets associated with hypothesised benefits in these areas may inadvertently be causing microcirculatory shut down in the systemic circulation and worsening outcomes. Due to the limitations of this study, further investigation is required to evaluate the role that adrenaline infusions have in this period of patient care.

## Author contributions

MS conducted the statistical analysis. PO acts as the guarantor for this article.

## Conflict of interest

The primary investigator (PO) works for SECAmb as a CCP.

## Ethics

This study was reviewed by the local NHS Research Ethics Committee (South West Central Bristol, 261365) and approved by the Health Research Authority and University of Hertfordshire prior to commencement.

## Funding

None.
